# Surveillance and Cleavage of Eukaryotic tRNAs

**DOI:** 10.3390/ijms16011873

**Published:** 2015-01-15

**Authors:** Cyrille Megel, Geoffrey Morelle, Stéphanie Lalande, Anne-Marie Duchêne, Ian Small, Laurence Maréchal-Drouard

**Affiliations:** 1 Institut de Biologie Moléculaire des Plantes, CNRS, Associated with University of Strasbourg, 12 rue du Général Zimmer, F-67084 Strasbourg Cedex, France; E-Mails: cyrille.megel@ibmp-cnrs.unistra.fr (C.M.); geoffrey.morelle@ibmp-cnrs.unistra.fr (G.M.); stephanie.lalande@ibmp-cnrs.unistra.fr (S.L.); anne-marie.duchene@ibmp-cnrs.unistra.fr (A.-M.D.); 2 Australian Research Council Centre of Excellence in Plant Energy Biology, the University of Western Australia, Crawley WA6009, Australia; E-Mail: ian.small@uwa.edu.au

**Keywords:** tRNA decay, tRNA quality control, sncRNAs, tRFs, stress response, gene expression regulation, tRNA endonucleases

## Abstract

Beyond their central role in protein synthesis, transfer RNAs (tRNAs) have many other crucial functions. This includes various roles in the regulation of gene expression, stress responses, metabolic processes and priming reverse transcription. In the RNA world, tRNAs are, with ribosomal RNAs, among the most stable molecules. Nevertheless, they are not eternal. As key elements of cell function, tRNAs need to be continuously quality-controlled. Two tRNA surveillance pathways have been identified. They act on hypo-modified or mis-processed pre-tRNAs and on mature tRNAs lacking modifications. A short overview of these two pathways will be presented here. Furthermore, while the exoribonucleases acting in these pathways ultimately lead to complete tRNA degradation, numerous tRNA-derived fragments (tRFs) are present within a cell. These cleavage products of tRNAs now potentially emerge as a new class of small non-coding RNAs (sncRNAs) and are suspected to have important regulatory functions. The tRFs are evolutionarily widespread and created by cleavage at different positions by various endonucleases. Here, we review our present knowledge on the biogenesis and function of tRFs in various organisms.

## 1. Introduction

Transfer RNAs (tRNAs) are among the most ancient RNAs in the world. Since their discovery by Hoagland and Zamenick in 1957 and the historical hypothesis of Francis Crick postulating the existence of an adapter molecule at the interface between the genetic code and protein, tRNA molecules are still “à la mode”. The first primary tRNA sequence [1] was followed by the determination of the characteristic cloverleaf secondary structure, and the L-shaped three dimensional tertiary structure was obtained in the seventies [2,3]. The sizes of tRNAs vary classically between 73 and 90 nucleotides. However, tRNAs from metazoan mitochondria can be much smaller. The world’s smallest armless tRNAs (42 nucleotides) were recently identified in the mitochondria of nematodes [4]. The first step in the production of a mature tRNA molecule is the synthesis of a primary transcript. In eukaryotes, RNA polymerase III transcribes tRNA genes and transcription stops after a series of U residues present a few nucleotides 3' of the tRNA sequence. The 5' leader and the 3' trailer sequences are removed by RNase P and RNase Z, respectively. The CCA 3' extremity is added by the tRNA nucleotidyl transferase. For a subset of tRNAs, introns located between positions 37 and 38 are removed through tRNA splicing. Splicing of tRNAs is a process including mainly two steps, the excision of the intron by an endonuclease and the ligation of two tRNA half-molecules. In yeast and plants the second step involves a single enzyme Trl1 catalyzing phosphodiesterase, kinase and ligase activities. In vertebrates, in addition to the yeast-like ligation pathway, a second pathway exists with a ligase that still needs to be identified (for a review see e.g., [5]). Multiple post-transcriptional modifications are also added all along the maturation of tRNA. To function in protein synthesis, tRNA is finally charged with an amino acid by its cognate aminoacyl-tRNA synthetase (e.g., [5]).

Transfer RNA molecules are among the most stable RNAs in a cell. Compared to mRNAs, with a half-life varying from minutes to a few hours, tRNA half-lives are more in the range of several days [5]. This extreme stability is due to a highly folded structure and the presence of numerous modifications. Nevertheless, quality control mechanisms exist to avoid the accumulation of non-functional tRNAs. In contrast to the many studies of mRNA decay [6,7,8,9], there has been less attention to the degradation of stable RNAs such as rRNAs or tRNAs, although they are the most abundant RNAs in the cell. Two major surveillance pathways have been uncovered that act in tRNA turnover in eukaryotes [10]. The degradation can occur at the level of pre-tRNA or mature tRNA species. The first tRNA degradation pathway involves the TRAMP (Trf4 (topoisomerase 1-related 4)/Air2 (Arginine methyltransferase-interacting RING finger protein 2)/Mtr4p (mRNA transport regulator 4 protein) Polyadenylation) complex and the nuclear exosome. The second tRNA degradation pathway is called RTD for Rapid tRNA Decay and involves cytoplasmic exonucleases. Our knowledge on the two pathways will be the topic of the first part of this review.

Beside their canonical role during protein synthesis, novel additional functions are regularly assigned to tRNAs (for reviews see [5,11,12,13]). In particular, they are involved in many processes of cellular metabolism. They can deliver amino acids not only for protein synthesis but also for cell envelope remodeling, antibiotic synthesis or tagging for proteolysis. Regulation of cell death by tRNA represents another important role. They can also be used as primers for viral reverse transcription. Moreover, uncharged tRNAs can act as stress sensors and as regulators of gene expression. Finally, small non-coding RNAs deriving from tRNAs potentially represent a new class of regulatory RNAs. Indeed, while in the tRNA surveillance pathways tRNA degradation is complete and no degradation products are expected to persist, relatively stable tRNA-derived RNA fragments (tRFs) of different sizes have been found in many different organisms. Half tRNAs are primarily created upon different stresses but shorter tRNA fragments of about 15–25 nucleotides are also abundant. Endonucleases responsible for tRNA or pre-tRNA slicing have been identified. Slicing can occur at different sites, in particular within the anticodon, D and T loops of the tRNA molecule. Potentially important functions have been ascribed to tRFs, including the inhibition of translation, or the induction of RNA silencing or apoptosis. In the second part of the review, we summarize our present knowledge on the population, biogenesis and function of tRFs.

## 2. Eukaryotic Transfer RNA (tRNA) Turnover and Quality Control

### 2.1. The Nuclear Surveillance Pathway

**Figure 1:**
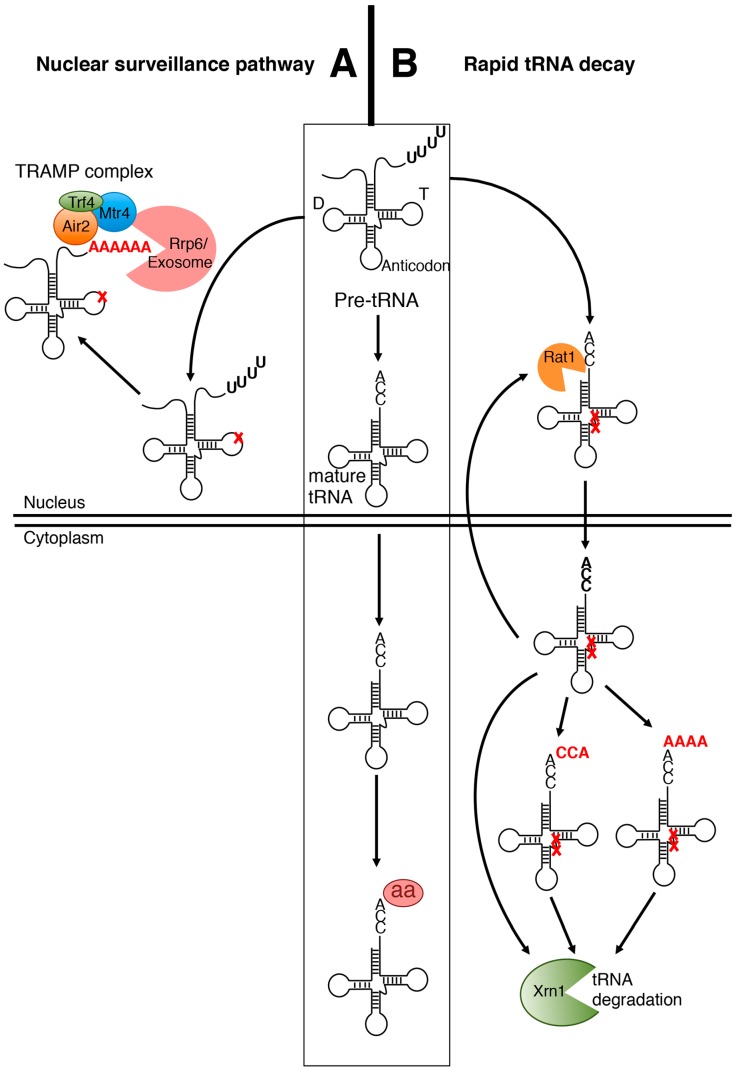
Overview of the two tRNA degradation pathways of *S. cerevisae.* This figure is adapted from [10]. (**A**) The tRNA nuclear surveillance pathway was primarily shown to act on pre-tRNAs lacking modifications (e.g., pre-tRNA_i_^Met^). Hypo-modified pre-tRNA is first polyadenylated by the TRAMP complex and then degraded by the exosome; (**B**) Degradation of mature tRNAs through the rapid tRNA decay (RTD) pathway. RTD was shown to act on hypo-modified tRNAs (marked with red crosses) (e.g., tRNA^Val(AAC)^). The major components are depicted. The implication of the tRNA-nucleotidyl transferase (CCAse) enzyme in the RTD pathway is also presented [33]. A, CCA triplet or a short poly(A) tail (AAAA) depicted in red can be added by the CCAse enzyme.

The nuclear surveillance pathway (Figure 1A) was discovered in temperature-sensitive yeast (*Saccharomyces cerevisiae*) strains lacking the tRNA methyl transferases (Trm) Trm6 or Trm61 (also named Gcd10 or Gcd14 for General control non-derepressible proteins 10 and 14) the two essential subunits of the methyltransferase responsible for tRNA m^1^A modification. In these strains, the precursor of initiator tRNA^Met^ (pre-tRNA_i_^Met^) lacks m^1^A_58_ and is subject to rapid turnover [14,15]. *In vivo* genetic analyses provided evidence that as a first step towards its degradation, the pre-tRNA_i_^Met^ is polyadenylated at its 3' end by the TRAMP complex. This complex was first shown to be required for polyadenylation and degradation of rRNA and snoRNA (small nucleolar RNA) precursors as part of a post-transcriptional quality control mechanism [16]. It is constituted by Trf4, a poly(A) polymerase, Mtr4, a RNA helicase, and Air2, a zinc knuckle protein interacting with Rrp6 (Ribosomal RNA Processing subunit 6), the 3' exoribonuclease of the nuclear exosome. Following polyadenylation by Trf4, pre-tRNA_i_^Met^ is degraded from the 3' end by Rrp6 [10,17,18,19,20,21]. Both the TRAMP and exosome complexes are localized in the nucleus. As described above in yeast, the pre-tRNA nuclear surveillance pathway is implicated in the degradation of defective hypomodified pre-tRNA_i_^Met^. However, how a tRNA is recognized or not as a substrate to be adenylated and degraded remains largely unknown. The lack of m^1^A_58_ is *per se* not sufficient to explain why the pre-tRNA_i_^Met^ is rapidly degraded as other tRNAs lacking the same modification are not degraded [18]. The most reasonable hypothesis is that the nuclear surveillance pathway recognizes aberrant mis-folded tRNA tertiary structure. *In vitro* data suggest that other tRNAs can be TRAMP substrates, but this remains to be demonstrated *in vivo* [16,22].

Different end-matured but unspliced intron-containing pre-tRNAs were also shown to be TRAMP targets [23]. Indeed, the authors showed that such targets are enriched in *trf4*Δ cells, a phenotype accentuated in *Rrp*6∆ cells. These results are linked to the complexity of the 3'-end maturation of pre-tRNAs, maturation that can be achieved by two different pathways [21,24]. In the first major pathway, the La protein (Lhp1p in yeast) binds the short stretch of uridines (Us), (at least 3) present at the 3' extremity of eukaryotic pre-tRNA, stabilizes the conformation and protects the extremity from exonucleases [25]. After removal of the 5' extension by RNAse P, the 3' tail is removed by an endonuclease such as RNAse Z. In the second pathway, Lhp1p does not bind or is released from the polyU tail. This allows Rex1p, a non-exosome exonuclease, to trim the 3' trailer up to a base-paired region between the 5' leader and the 3' trailer. Following cleavage of the 5' extremity by RNAse P, Rex1p or other exonucleases mature the 3' extremity and the CCA sequence can be added. Considering these two pathways, it is likely that a fraction of intron-containing pre-tRNAs folding into alternative abnormal structure fails to bind Lhp1p. This aberrant structure may slow the rate of end maturation, and the competition between Lhp1p and Rex1p will allow additional exonucleolytic nibbling and/or TRAMP polyadenylation [26]. Altogether there is a strong interplay between maturation pathways that functional tRNAs undergo and quality control pathways that degrade abnormal RNAs. This also implies strong competition between the La protein Lhp1p, the exonuclease Rex1p and the TRAMP complex [23].

### 2.2. The Rapid tRNA Decay (RTD) Pathway

While the nuclear surveillance pathway acts primarily on pre-tRNAs, mature tRNAs also undergo quality control surveillance. As for the nuclear surveillance pathway, the first evidence of the existence of a pathway called rapid tRNA decay (RTD) came from yeast mutant strains lacking enzymes involved in tRNA modifications. The temperature sensitive *trm8*∆*trm4*∆ yeast mutant strain presents a severe growth defect. In this strain, the mature tRNA^Val(AAC)^ lacks both 7-methylguanosine (m^7^G_46_) and 5-methylcytidine (m^5^C) and is rapidly deacylated and degraded at 37 °C [27]. Similarly, degradation of several other mature tRNAs lacking different combinations of modifications was also observed. For instance, in *trm44*∆*tan1*∆ or in *trm1∆trm4∆* yeast cells lacking 2'-*O*-methyluridine (Um_44_) and 4-acetylcytidine (ac^4^C_12_) or di-methylguanosine (m_2,2_G_26_) and 5-methylcytidine (m_5_C) modification enzymes respectively, both mature tRNA^Ser(CGA)^ and tRNA^Ser(UGA)^ are progressively lost at 37 °C [28,29]. As modifications are known to be involved in thermal stability of tRNAs (e.g., [30,31]), this supports the idea that this pathway may act on a wide variety of unstable or non-functional tRNA species.

The hypothesis is that, in strains lacking tRNA modification enzymes, under normal conditions most tRNAs are correctly folded and functional, but after the temperature switch the hypo-modified tRNAs are destabilized and trigger RTD. It has also been shown that the RTD pathway can act on tRNAs bearing mutations that are deleterious for the stability of the acceptor and T-stems. Fully modified tRNAs bearing these mutations are degraded, thus demonstrating that surveillance by the RTD pathway does not necessarily depend on tRNA modifications but rather on the structural integrity of the RNA [32].

The loss of hypo-modified tRNAs is not dependent on Trf4 or Rrp6, implying that the TRAMP complex and the nuclear exosome are not implicated. Rather, other protein components belonging to the RTD pathway were genetically identified (Figure 1B). In the *trm8*∆*trm4*∆ yeast mutant strain, the degradation and loss of aminoacylation of tRNA^Val(AAC)^ is mediated by Met22 (Methionine-requiring protein 22) and the two 5'–3' exonucleases Rat1 (Ribonucleic-acid-trafficking protein 1) and Xrn1 (Exoribonuclease 1) [34]. Deletion of Met22 or Rat1/Xrn1 in a *trm8∆trm4∆* or *trm44∆tan1∆* strain rescues the temperature-sensitive phenotype entirely by preventing the degradation of several tRNAs in yeast [34], and in humans [35]. The degradation by the nuclease Xrn1 is likely due to the instability of the acceptor stem, which exposes the 5' end of the tRNA molecule [32]. Rat1 is localized in the nucleus, thus suggesting the need to re-import deleterious tRNAs into this compartment to allow their degradation by this endonuclease. However, the requirement of the retrograde tRNA nuclear import pathway using importin Mtr10 (mRNA transport regulator 10), [36] needs to be demonstrated. Met22 is a phosphatase not directly implicated in the RTD pathway. Rather, when function of Met22 is lost, accumulation of its metabolite substrate adenosine 5',3'-biphosphate (pAp) inhibits Rat1 and Xrn1 [27,34,37,38]. Interestingly, the inhibition of plant exoribonucleases by pAp has also been observed and it is the plastidial enzyme SAL1 (SAL for salt tolerance) which plays the role of Met22. Whether the degradation of tRNAs is prevented in *sal1* mutants, remains to be established [39].

Furthermore, in the *trm8∆trm4∆* strain, the stability of hypo-modified tRNA^Val(AAC)^ is restored when the genes *TEF1* and *VAS1* encoding the elongation factor eEF1A and valyl-tRNA synthetase respectively are over-expressed [29,40]. These two proteins interact directly with the tRNA molecules: tRNA^Val^ is aminoacylated by valyl-tRNA synthetase, bound to eEF1A and delivered to ribosomes. Thus, it is likely that an increase in the levels of proteins directly interacting with imperfectly folded hypo-modified tRNAs can protect them from degradation by competing with ribonucleases such as Rat1 and Xrn1. This shows that components of the RTD and of the translation machinery actively interact to either deliver charged tRNA molecules to ribosomes or degrade them. Another category of proteins able to limit the RTD process corresponds to proteins affecting global tRNA transcription. In the *trm8∆trm4∆* strain, over-expression of Maf1, the global negative regulator of polymerase III (polIII) [41,42] results in lower degradation of the hypomodified tRNA^Val(AAC)^ [40]. Similarly, in the same mutant strain, point mutations in the RNA polIII Rpc128 subunit (RNA closed complex subunit 128) or decreased expression of Rpc17, another polIII subunit, leads to a decrease in tRNA transcription and to tRNA^Val(AAC)^ stabilization.

The presence of a CCA triplet at the 3' extremity of tRNAs is essential for their charging with cognate amino acids. The tRNA nucleotidyltransferase, also called CCA-adding enzyme or CCAse, post-transcriptionally adds the CCA triplet at the 3' end of tRNAs, a sequence not genome-encoded in eukaryotes [43,44,45].

In addition, this enzyme can also add CCA to the 3' ends of tRNA-like transcripts (e.g., [46]). Wilusz and collaborators [33] provided evidence that the CCA-adding enzyme is able to selectively mark unstable tRNAs (e.g., hypo-modified tRNAs or tRNAs lacking integrity of the acceptor stem) or tRNA-like small RNAs for degradation by adding the CCACCA sequence. The CCACCA addition requires an isomerization step and the presence of two Gs at the 5' extremity of the RNA molecule. This creates a long 3' single-stranded RNA tail and likely allows access by the exonucleases Rrp44 (Ribosomal RNA processing subunit 44) and Xrn1 (Exoribonuclease 1), which then initiate tRNA degradation. The proposed model also explains why some tRNAs are sensitive to RTD whereas others, similarly hypo-modified, are not. The latter do not contain two guanosines (Gs) at their 5' extremity, thus precluding the isomerization step required for the double CCA addition. Finally, the authors also observed an increased number of tRNAs with short poly(A) tails added to the CCA motifs, another way to flag unstable RNAs for degradation.

In other eukaryotes, tRNA degradation pathways have not been studied yet. Many of the enzymes involved in either the nuclear surveillance pathway or the RTD pathway in yeast exist in plants and mammals. Therefore, it is likely the same tRNA quality control pathways are present in organisms from yeast to vertebrates [47], although it remains to be demonstrated.

## 3. tRNA-Derived Fragments (tRFs): A New Class of Small Non-Coding RNAs

Since the discovery in 1993 of small non-coding RNAs (sncRNAs) in *Caenorhabditis elegans* [48], many classes of sncRNAs (15–40 nt long) with various regulatory functions have been identified in a multitude of organisms of the eukaryotic kingdom [49]. The most well-known are the siRNAs (small-interfering RNAs), miRNAs (microRNAs) and piRNAs (piwi-interacting RNAs).

Thanks to the increasing use of high-throughput sequencing technologies many more classes of sncRNAs are emerging [50,51]. Among them, short tRNA-derived RNA fragments (tRFs) of 15–28 nt have been found in sncRNA libraries of various organisms. Eukaryotic tRFs were first characterized in the human HCT (Human Lymphocyte T) 116 cell line [52]. Since then, they have been described in all domains of life (e.g., [53,54,55,56,57,58,59,60,61,62,63,64]). Longer tRFs of about 30–40 nt in size are also reported in the literature. In eukaryotes, they were discovered in evolutionary divergent organisms such as yeast, protozoans, plants and metazoans (e.g., [54,56,60,61,65,66,67,68,69,70,71,72]). Most of the time, RNA fragments with a length exceeding 30 nt are excluded from sncRNA libraries and this second category of tRFs was mainly identified by northern blots.

tRFs are present in very low amounts within cells as compared to the high steady-state levels of tRNAs from which they derive. Nevertheless, it is now suspected that tRFs are not just degradation products but may be implicated in important regulatory and biological processes. In the following paragraphs, the different classes of tRFs will be presented in more detail.

tRFs are classified according to the region of the tRNA or of the pre-tRNA from which they derive. Three classes of short tRFs have been described. They originate from (i) the 5' extremity of mature tRNA after cleavage in the D region; (ii) the 3' extremity of the mature tRNA (meaning the CCA end is present) after cleavage in the T region and (iii) the 3' trailer of pre-tRNA after cleavage by RNAse Z.

As the data were obtained by several teams concurrently, various nomenclatures have been proposed (Table 1). For example, tRFs corresponding to RNA fragments derived from the 5' end of mature tRNA after cleavage in the D region were named 5' tRF or tRF5 (e.g. [73]), those deriving from the 3' end were named 3' CCA tRF or tRF3 [61,74]. These tRFs were recovered in sncRNA libraries because of their size. The remaining tRNA fragments (*i.e.*, fragments of around 40–60 nt) would be considered as long tRFs. Due to their size, these RNA fragments cannot be retrieved from the sncRNA libraries, but their existence cannot be excluded. Indeed, using northern experiments, such RNA fragments are present in total *Arabidopsis thaliana* RNA extract (personal communication, Laurence Maréchal-Drouard). Taking into account the existence of a more complex population of tRFs than presently described, we propose a novel nomenclature where each tRF is named according to the extremity of the mature tRNA and the cleavage region from which it is generated (Table 1). For instance, tRF-5D represents a tRF generated from the 5' extremity of a tRNA after cleavage in the D region. The complementary fragment is then called tRF-3D. To this nomenclature it is possible to add the amino acid and the anticodon specifying the corresponding tRNA. For example, tRF-3T (His-GUG) means tRF generated from 3' extremity of tRNA^His(GUG)^ after cleavage in the T region. Most tRFs described so far originate from nuclear-encoded cytosolic tRNAs. However a few examples of tRFs deriving from plastidial or mitochondrial tRNAs have been described [75,76]. It is thus also important for the future to add this information by adding c (cytosolic), p (plastidial) or m (mitochondrial) when necessary. In mammals, tRFs deriving from the 3' trailer of pre-tRNAs were also identified. These tRFs, called 3' U tRF or tRF1, match to the 3' trailer of precursor tRNA molecules [52,57,77]. They are released upon RNAse Z cleavage and end at the level of the short stretch of U residues where polymerase III is released. They do not derive from mature tRNAs but rather from precursor transcripts, and we propose to rename them pre-tRF-3U.

**Table 1:** General nomenclature of tRNA-derived fragments (tRFs). Mature tRNAs and pre-tRNAs can be cleaved at different positions to produce various tRFs. Different names have been proposed according to their size, function and position [13,50,66,78]. We now propose a general nomenclature where the letter corresponds to the extremity of the tRNA and the number to the cleavage site. The localization of the tRFs are drawn either in red or in black on the tRNA molecule and their names have the same color code.

tRFs	Yamasaki, 2009; Anderson, 2014	Raina, 2014	Lee, 2009	General Nomenclature	
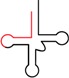	5'-tRF	5'-tRF	tRF5	tRF-5D	tRF-3D
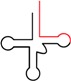	3' CCA tRF	3' CCA tRF	tRF3	tRF-5T	tRF-3T
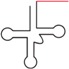		3' U tRF	tRF1		pre-tRF-3U
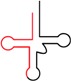	5'-halves (5'-tiRNAs)	5' tRNA halves		tRF-5A	tRF3A
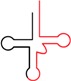	3'-halves (3'-tiRNAs)	3' tRNA halves		tRF-5A	tRF3A

Another category of tRFs corresponds to mature tRNA halves generated through cleavage in the region of the anticodon. From a single tRNA, two fragments of about 30–40 nt are thus generated, and correspond to the 5' and 3' tRNA halves respectively (Table 1). This category of tRFs is generally produced in response to a multitude of stresses (e.g., amino acid starvation, phosphate starvation, oxidative stress) (e.g., [61,72,78,79,80]). In mammals, they were therefore called tiRNAs (tRNA-derived stress-induced RNAs) or also 5' tRNA halves (for reviews see [13,81]). This category of tRFs has been found in all kingdoms of life: bacteria, protozoa, fungi, plants and animals and seem to be a universal marker of stress (e.g., [56,60,61,65,66,67,69,70,71,72]). Nevertheless, it is worth to mention that not all stresses induce tRNA cleavage. For instance, in yeast cells undergoing amino acid or glucose starvation or UV irradiation, no increase of this category of tRFs was observed [61,69,82]. Furthermore, tRFs corresponding to tRNA halves were also shown to be present *in vivo* in the absence of stress. [61,72,80]. The anticodon region is the most accessible part of a tRNA molecule for either mechanical or enzymatic cleavages. The presence of these RNA fragments is thus not unexpected and whether some of them play regulatory functions still remain to be established. Finally, as the 5' tRNA half and the 3' tRNA half do not have similar functions (see below for more details), it is essential to distinguish them. Thus, as above for short tRFs, we propose a similar nomenclature where tRF-5A and tRF-3A will represent the 5' tRNA half and the 3' tRNA half of a mature tRNA after cleavage in the region of the anticodon.

Finally, the last known example of tRFs corresponds to 5' leader-exon tRNA fragments. In mice lacking CLP1 (Cleavage and Polyadenylation factor I subunit 1, an RNA kinase implicated in tRNA splicing in mammals), accumulation of such tRFs deriving from an aberrant splicing of intron-containing pre-tRNAs^Tyr^ was observed [83]. Interestingly the authors showed that this category of tRFs sensitizes cells to oxidative-stress induced p53 activation and p53-dependent cell death but the exact molecular mechanism remains to be characterized.

## 4. Biogenesis of tRF: Which Endonuclease for Which Job?

Among the first identified tRNA endonucleases were bacterial Colicin E5 and Colicin D (Table 2). In *E. coli*, both enzymes cleave specific tRNAs in the anticodon loop. The major consequence is the arrest of protein synthesis leading to cell death [65,66]. Colicin E5 endonuclease recognizes tRNAs containing a queuine (Q) residue at the first position of the anticodon and cleaves between position 34 and 35. Colicin D endonuclease cleaves the four tRNA^Arg^ isoacceptors between positions 38 and 39. Another study illustrates an anticodon endonuclease activity. During phage T4 infection, suppression of EcoprrI DNA restriction activity allows the PrrC (Pre-mRNA cleavage complex II) protein subunit to cleave tRNA^Lys^ between position 33 and 34 [84,85].

In eukaryotes, tRNA endonucleases have also been identified. Zymocin (γ-subunit) of the dairy yeast *Kluyveromyces lactis* induces G1 phase arrest of sensitive yeast cells like *Saccharomyces cerevisiae* by cleavage in the anticodon loop between position 34 and 35 of tRNA^Lys^ and tRNA^Gln^ [86]. These tRNAs are unusual in that they carry a 5-methoxycarbonylmethyl-2-thiouridine (mcm^5^) residue at the wobble position. The absence of mcm^5^ group drastically reduces the cleavage efficiency suggesting that post-transcriptional nucleotide modifications are important for a proper enzyme-substrate interaction and cleavage. *S. cerevisiae* also possesses its own tRNA endonuclease called Rny1 (Ro-associated Y1). Rny1 belongs to the RNase T2 family, and is able to cleave tRNAs within the anticodon loop [87,88]. Rny1 is normally localized in the vacuole [87]. Where this ribonuclease cleaves tRNAs is still under debate. Thompson and Parker showed that, after oxidative stress, Rny1 can be released from the vacuole to the cytoplasm and that this release correlates with tRNA cleavage [87]. In contrast, Luthala and Parker presented evidence that tRNAs are targeted to vacuoles and that Rny1 cleaves tRNAs in this compartment. They proposed that this enzyme might participate in a form of tRNA ribophagy [87,88]. Recently, in agreement with these data, work from Huang *et al.* demonstrated that vacuole-localized Rny1 is responsible for the degradation of unidentified RNA in nitrogen starving conditions [89]. RNase T2 enzymes are also implicated in tRNA cleavage in the protozoan *Tetrahymena thermophila* [90]. Three endonucleases induced during amino acid starvation are expressed from homologous genes (*RNT2A*, *RNT2B* and *RNT2C*) and contribute to the production of tRNA halves by cleavage in the anticodon loop. In mammals, several endonucleases have been identified as capable of tRNA cleavage. They are involved at different stages of tRNA processing and exert their activities at different sites. A study performed by Lee *et al.* [52] showed that the RNase Z, ELAC2 (elaC ribonuclease Z2), produces pre-tRF-3U from tRNA precursors. For instance, the pre-tRF-3U called tRF1001 is generated by cleavage at the 3' end of the precursor of tRNA^Ser(TGA)^. Dicer, a double-stranded RNA-specific endonuclease playing a central role in RNA silencing pathway, is implicated in the production of a specific tRF-5D deriving from tRNA^Gln(CUG)^ after cleavage in the single-stranded D loop [73]. Other studies also revealed the role of Dicer in the production of pre-tRF-3U and tRF-3T deriving respectively from either the 3' extremity of pre-tRNA^Ile^ [91] or from the 3' extremity of mature tRNA^Glu^ [57]. These results suggest that some tRNAs, tRNA halves or pre-tRNAs can adopt alternative secondary structures to form a dsRNA enabling Dicer cleavage. However, Dicer does not seem to be implicated in the production of all tRF-5D or tRF-3T and other still unknown endonucleases are likely required. Finally, a vertebrate-specific endonuclease called angiogenin (ANG) has been shown to cleave tRNAs in the anticodon loop generating the long tRFs, tRF-5A and tRF-3A [69]. ANG belongs to the RNase A family and is induced by a large variety of stresses such as oxidative stress, UV irradiation, heat shock or viral infection. The particularity of ANG is that it was first identified as a tumor angiogenic factor acting in the nucleus during cell growth and proliferation. Under normal conditions, ANG localizes primarily to the nucleus and to a lesser extent to the cytoplasm where it is associated with RNH1 (RNase H inhibitor 1), its inhibitor [92]. Under stress conditions, it has been proposed that either the nucleus-localized ribonuclease is exported to the cytoplasm or that the cytoplasmic-localized ANG is dissociated from RNH1 to produce tRNA halves from mature tRNAs [93]. This enzyme seems to play a strong role in a cytoprotective stress response program. ANG catalytic activity is also essential to promote stress granule assembly through tRF production [94]. Importantly, beside the ability to cleave in the anticodon region of tRNAs, ANG plays a crucial role in cleaving the 3'-CCA extremity of tRNAs upon oxidative stress [95]. Indeed, the authors showed that the cleavage by ANG at the level of the CCA extremity occurs much faster than the cleavage in the anticodon region. Eliminating the CCA ends of tRNA molecules represents a very rapid and efficient way to inhibit translation. This process is reversible as the CCA extremities can be rapidly repaired by tRNA-nucleotidyl transferase. This cleavage at the 3' ends of tRNAs reflects the substrate specificity of ANG which preferentially targets single-stranded RNA at the level of a CA-motif. While ANG can cleave all tRNAs at their 3' end, only a subset of tRNAs (*i.e.*, those with a CA-motif in the anticodon region) will be cleaved in the anticodon loop to generate tRFs. These data suggest that the primary function of ANG is the cleavage of the CCA 3' ends of tRNAs.

**Table 2:** List of endonucleases capable of cleaving tRNAs or pre-tRNAs.

Organisms	Endonucleases	tRNA Specificities	Cleavage Sites	References
*E. coli*	Colicin E5	Queuine-containing tRNA; tRNA^Arg^	Anticodon loop	[65]
	Colicin D		Anticodon region	[66]
	PrrC	tRNA^Lys^	Anticodon loop	[84]
*K. lactis*	Zymocin	tRNA^Lys^; tRNA^Gln^	Anticodon loop	[86]
*S. cerevisiae*	Rny1	No	Anticodon loop	[87]
*T. thermophila*	Rnt2A–C	No	Anticodon loop	[90]
Mammals	ELAC2	pre-tRNA^Ser^	3' end	[52]
	Dicer	tRNA^Gln^	D loop	[73]
		tRNA^Glu^	T loop	[57]
		pre-tRNA^Ile^	3' end	[91]
	Angiogenin	No	Anticodon loop	[69]

Concerning short tRFs, with the exception of Dicer in a very few instances, mostly nothing is known concerning the identification and the location of the endonucleases responsible for the cleavage in the D or T regions of mature tRNAs. Similarly, the subcellular location of these tRFs is largely unknown. In the epimastigote form of *Trypanosoma cruzi*, the subcellular location of tRFs has been reported by [55]. Using Fluorescent *In Situ* Hybridization (FISH) experiments, the authors not only observed the recruitment of tRNA-halves to specific cytoplasmic granules but also showed that depending on the nature of the tRFs (tRF-5A or tRF-3A), the RNA fragments localize in distinct cytoplasmic granules. Furthermore, Reifur and collaborators [96] reported a differential location of tRFs depending on the developmental stage of *T. cruzi*. In the epimastigote form, tRFs localize to posterior cytoplasmic granules whereas in the metacyclic form, they are evenly distributed in the cytoplasm. These studies suggest that location of short tRFs may have an important biological significance since they localize in specific structures or compartments where they may interact with particular partners for as yet undescribed functions.

## 5. The Emerging Roles of tRFs

**Figure 2:**
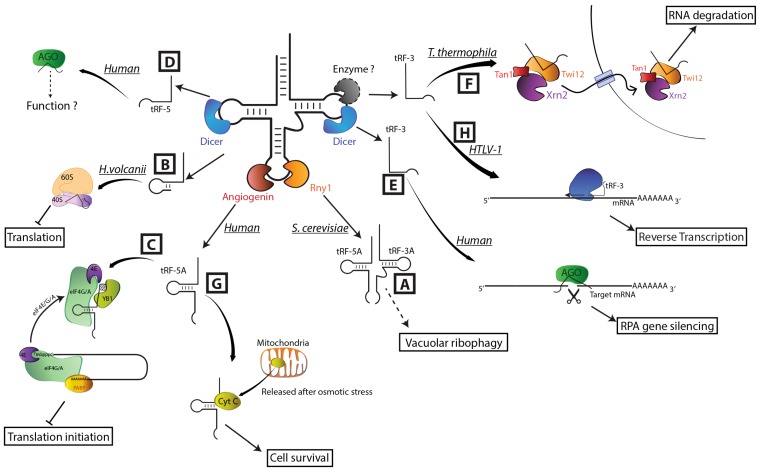
Summary of our knowledge on the major identified molecular functions assigned to tRFs. References for the functional implications of tRFs are the following: (**A**) [89]; (**B**) [56]; (**C**) [97]; (**D**) [73]; (**E**) [74]; (**F**) [98]; (**G**) [99]; (**H**) [100]. Straight arrows indicate the generated tRFs and bent arrows their functions. Dashed arrow indicates potential functions.

Under nutrient starvation (e.g., phosphate or nitrogen starvation), it is essential to recycle constituents present in stable macromolecules for the cell to survive. For instance, RNA degradation constitutes a very efficient way to deliver phosphate and nitrogen upon starvation. Ribosomal RNAs and tRNAs are the most abundant RNAs and represent, via their degradation, major sources for the recycling of these components. Indeed, in *S. cerevisiae*, the existence in vacuoles of a nitrogen starvation-induced ribophagy pathway involving Rny1 has been identified (Figure 2A), [89]. Thus tRFs are likely to be products of the earliest stages of ribophagy, on the route to providing needed phosphate and nitrogen to the cell. In plants, in response to phosphate deficiency, tRF-5s accumulate at a high level [58,71]. Whether the only fate of this pool of sncRNAs is to provide nutrients to the cell via their complete degradation of whether some of them play other molecular functions is an open question. Evidence of additional functions for some tRFs is emerging in many organisms. Our present knowledge is summarized below and Figure 2 likely represents only the tip of the iceberg.

Indeed, one of the best-characterized functions of tRFs is their implication in the inhibition of protein synthesis. Several studies have shown that sncRNAs deriving from tRNAs are able to inhibit translation independently of any RISC (RNA-induced silencing complex) function. In the Archaeabacteria *Haloferax volcanii* (Figure 2B), a specific 26 nt long tRF-5D generated from tRNA^Val(GAC)^ targets the small ribosomal subunit reducing protein synthesis by interfering with peptidyl transferase activity [56]. In humans, as already described above, the most efficient and fastest way for ANG to inhibit translation is to eliminate the 3'-CCA end of tRNAs [95]. Nevertheless, in human U2OS cells (Figure 2C), inhibition of initiation of translation has also been observed through the action of very specific ANG-induced tRNA halves [97]. Interestingly, only tRF-5A and not tRF-3A are able to repress translation. They also showed that only tRF-5D (Ala) and tRF-5D (Cys) are strong inhibitors of translation. These fragments are able to interact with the translational silencer YB-1 to inhibit translation by displacing eukaryotic initiation factor 4F (eIF4F) complex from capped mRNAs or eIF4G from uncapped mRNAs. Two structural features of these tRFs are required to inhibit translation. First, the dimension of the stem-loop of the D region is important. Second, a 5'-terminal oligoguanine (TOG) motif must be present. Indeed, Ivanov *et al.* demonstrated recently that TOG forms a G-quadruplex structure essential for translation inhibition [101].

Another aspect suggesting that tRFs can be implicated in the regulation of gene expression comes from the analysis of small RNAs associated with Argonaute (AGO) complexes [57,76,102,103]. Dicer-generated tRF-5Ds are only poorly associated with AGO complexes (Figure 2D) [73]. In contrast, pret-RF-3Us were found to be preferentially associated with AGO3 and AGO4 in HEK293 cell lines [57]. Moreover, AGO1-associated tRFs deriving from several human tRNAs [76] and from the 5' end of tRNA^Glu^ in *Schizosaccharomyces pombe* were also uncovered [104]. So far, the only identified target for a tRF-AGO complex was shown in mature B cells (Figure 2E). In this case, a Dicer-dependent tRF-3T deriving from tRNA^Gly(GCC)^, is bound to AGO and represses RPA1 (Replication Protein A 1) , a gene implicated in DNA replication and repair. This is the first evidence that a tRF can play the role of a miRNA [74]. In plants, association of tRFs with several AGO proteins was also observed [105]. At least two hypotheses can be given concerning tRF functions in AGO complexes: (i) Loaded in RISC the tRFs can target a mRNA bearing the antisense sequence to modulate gene expression; (ii) In cooperation with AGO proteins, tRFs compete with the original small RNAs for loading in the RISC complex, repressing therefore the whole RNA silencing pathway. Still related to silencing, work in *Drosophila melanogaster* reported that the siRNA pathway can be affected by heat-shock-induced tRFs. Indeed, the authors showed that tRFs are able to inhibit Dicer-2 activity on long dsRNA in a *dnmt2* mutant background [106].

In *T. thermophila* (Figure 2F), the implication of tRFs in RNA metabolism has also been demonstrated. A detailed study by Couvillion *et al.* showed that tRF-3Ts interact with Twi12, a Piwi protein required for vegetative growth. This is the only Piwi Argonaute protein (out of eight) essential for cell survival. It is specialized in the loading of tRFs and these tRFs are essential for the nuclear RNA decay pathway. Indeed, the tRF-Twi12 complexes recruit Tan1 (Twi-associated novel 1) and Xrn2 (exoribonuclease 2) ribonucleases forming a TXT (Twi12/Xrn2/Tan1) complex. The TXT complex is then imported into the nucleus and stimulates Xrn2 exonuclease activity engaged in RNA processing and degradation pathways [98,107,108].

Further research has established a link between ANG-induced tRNA halves and particular functions. In the case of RSV (Respiratory Syncytial Virus) infection, Wang and collaborators found that an ANG-dependent tRNA half generated from tRNA^Glu(CTC)^ represses target mRNA in the cytoplasm and stimulates RSV replication [62]. Work from Saikia *et al.* [99] demonstrated that hyperosmotic stress leads to the accumulation of ANG-induced tRNA halves in the cytoplasm of mouse embryonic fibroblasts (Figure 2G). They showed that specific tRF-5As or tRF-3As interact with cytochrome *c* released from mitochondria to form a ribonucleoprotein complex. This complex interferes with the formation of the apoptosome by preventing the association of cytochrome *c* with Apaf-1, the apoptotic protease activating factor 1. Altogether this study demonstrates the cytoprotective effect of angiogenin via the production and accumulation of tRNA halves. Another recent function assigned to a tRF is as a primer for reverse transcriptase (Figure 2H). A tRF-3T (Pro) was shown to be capable of priming HTLV-1 (Human T-cell leukemia/lymphoma virus type 1) reverse transcriptase, thus suggesting an important role of some tRFs in viral infection [100].

Not only tRFs deriving from mature tRNAs may have important biological functions, but also those deriving from the 3' end of pre-tRNAs. A pre-tRF-3U called tRF-1001 deriving from the pre-tRNA^Ser(UGA)^ was found to be increased in various cancer cell lines and shown to be required for cell viability [52]. Several pre-tRFs-3U, including tRF-1001, were found associated with Argonaute proteins. This supports the idea that this category of tRFs plays a role in the global regulation of RNA silencing [57].

## 6. Conclusions and Perspectives

As key elements of the protein synthesis machinery, tRNAs and rRNAs must be, and are, highly stable macromolecules. Indeed, a rapid turnover of these structural non-coding RNAs would not be beneficial for the cell [32]. Nevertheless, and as described above, there are at least three main reasons to destroy a tRNA.

The first reason is linked to RNA quality control. If tRNA is unable to accurately and efficiently play its role in protein synthesis by correctly charging its cognate amino acid or to perform other additional functions more recently uncovered, its presence may be harmful for the cell. As described above, two major RNA degradation pathways involving exonucleases and acting at the level of either pre-tRNAs or mature tRNAs have evolved to avoid the presence of deleterious misfolded or hypo-modified tRNAs. Most of the work to unravel these pathways has been achieved in the yeast *S. cerevisiae*, but we can speculate that these tRNA surveillance pathways are likely conserved among evolutionarily divergent eukaryotes.

The second reason is to recycle key nutrients within the cell. It has been demonstrated that when eukaryotic cells are deprived of various nutrients, there is a retrograde nuclear import of tRNAs which accumulate in the nucleus. The reduction of tRNA abundance in the cytoplasm induces translational repression (for a review, see [109]). Another way for the cell to respond to nutrient deprivation is to degrade its own proteins and RNAs. Many data on tRFs report their accumulation under stress conditions. It is thus easily conceivable that stable RNAs such as rRNAs and tRNAs are degraded to provide resources to the cell. Quite surprisingly, there is little data in the literature on this aspect of tRNA turnover, and only a few studies on ribophagy are available.

The third reason to cut tRNAs into pieces is to use the pieces for other purposes. First assumed to be simply degradation products, there is now some evidence that specific tRFs play a role in regulating gene expression. The involvement of tRFs in a number of human diseases such as cancer or chronic diseases and in various environmental stresses is also emerging (see e.g., [13]). The functions attributed so far to tRFs are likely only the tip of the iceberg. Many questions still remain regarding both their biogenesis and their functions. From an evolutionary point of view, we can also wonder whether the same tRFs play identical roles in different eukaryotes. Finally, the role of modified nucleotides in producing functional tRFs also remains elusive and needs to be addressed in the future.
